# Quality control on digital cancer registration

**DOI:** 10.1371/journal.pone.0279415

**Published:** 2022-12-22

**Authors:** Stefano Guzzinati, Jessica Battagello, Emanuela Bovo, Maddalena Baracco, Susanna Baracco, Eva Carpin, Antonella Dal Cin, Anna Rita Fiore, Alessandra Greco, Giancarla Martin, Laura memo, Daniele Monetti, Silvia Rizzato, Carmen Stocco, Sara Zamberlan, Manuel Zorzi, Massimo Rugge

**Affiliations:** 1 Regional Epidemiology Service, Veneto Cancer Registry (RTV), Azienda Zero, Padova, Italia; 2 Department of Medicine DIMED, Surgical Pathology & Cytopathology Unit, University of Padova, Padova, Italia; Fondazione IRCCS Istituto Nazionale dei Tumori, ITALY

## Abstract

Population-based cancer registration methods are subject to internationally-established rules. To ensure efficient and effective case recording, population-based cancer registries widely adopt digital processing (DP) methods. At the Veneto Tumor Registry (RTV), about 50% of all digitally-identified (putative) cases of cancer are further profiled by means of registrars’ assessments (RAs). Taking these RAs for reference, the present study examines how well the registry’s DP performs. A series of 1,801 (putative) incident and prevalent cancers identified using DP methods were randomly assigned to two experienced registrars (blinded to the DP output), who independently re-assessed every case. This study focuses on the concordance between the DP output and the RAs as concerns cancer status (incident *versus* prevalent), topography, and morphology. The RAs confirmed the cancer status emerging from DP for 1,266/1,317 incident cancers (positive predictive value [PPV] = 96.1%) and 460/472 prevalent cancers (PPV = 97.5%). This level of concordance ranks as “optimal”, with a Cohen’s K value of 0.91. The overall prevalence of false-positive cancer cases identified by DP was 2.9%, and was affected by the number of digital variables available. DP and the RAs were consistent in identifying cancer topography in 88.7% of cases; differences concerned different sites within the same anatomo-functional district (according to the International Agency for Research on Cancer [IARC]) in 9.6% of cases. In short, using DP for cancer case registration suffers from only trivial inconsistencies. The efficiency and reliability of digital cancer registration is influenced by the availability of good-quality clinical information, and the regular interdisciplinary monitoring of a registry’s DP performance.

## Introduction

The Veneto Tumor Registry (Registro Tumori del Veneto [RTV]) was established in 1989, and has been registering all malignancies (excluding non-melanoma skin neoplasia) occurring in the region’s population of 4,900,000 since 2017 [1–3].

The data prompting digital cancer case registrations come from three main sources: i) pathology reports (PRs), based on the Italian pathology coding system, that are transferred digitally to the RTV via a dedicated data flow [4]; ii) hospital discharge records (HDRs), provided by the regional public health system, coded according to ICD-9-CM system [5]; and iii) death certificates (DCs), coded according to the ICD-10 [6], provided by the regional registry of cause of death. All data undergo quality control and transcoding to ICD-10.

Incident malignancies are identified by digital processing (DP) using dedicated software (SAS) [7, 8]. The algorithm adopted applies a set of specific decision-guiding criteria and automatically recognizes cancer cases recorded in pathology reports (PRs), hospital discharge records (HDRs) and/or death certificates (DCs). Because of the possible occurrence of inconsistent information among the above-mentioned source, about 50% of digitally-identified (putative) incident cancers are rejected. DP also rejects any multiple primary malignancies unless they are defined as metachronous by a random forest classifier based on certain predictive variables, such as chemo- or radiotherapies [9]. This rejection triggers a registrar’s assessment (RA), which involves specialized cancer registrars consulting all the available clinical-demographic records. Registrars accept or reject each case on the strength of supplementary information obtained by consulting imaging or endoscopy reports, A&E reports, patients’ clinical records, and so on. A subgroup of “putative” cancers may still need a collegial assessment by a team of registrars, epidemiologists, and pathologists with elective oncology expertise. The RA can therefore be confidently taken as the gold standard of cancer case registration.

Consistently with national (Italian Association of Tumor Registries [AIRTUM]) and international (International Agency for Research on Cancer [IARC]) guidelines, the following variables are recorded for all cases registered: a) demographics (date of birth, sex); b) place of residence (municipality at the time of diagnosis); c) date of first cancer diagnosis (d/m/y); d) topography and morphology, coded according to the International Classification of Diseases for Oncology (ICD-O-3) [10] and transcoded to the ICD-10; e) record(s) on which the cancer diagnosis is based [11], ranked from the most to the least reliable, i.e., from surgical pathology/cytology reports to clinical reports (including clinical pathology, imaging documents, etc.), to death certificates (DC) “only”; f) patient’s status (alive, or dead); and g) date of last available follow-up (d/m/y).

The present study aims to test the quality of the digital cancer registration process adopted by the RTV. Taking RAs for reference, we identify the proportions of: i) cases erroneously identified as cancer by DP (i.e., false-positives); ii) prevalent cancers mistakenly classified as incident cases; and iii) digitally misclassified metachronous malignancies.

## Materials and methods

### The digital cancer registration process

The DP method applies about fifty decision-guiding criteria to the available sources of data (PRs, HDRs and DCs) for each patient. These criteria include: a) cancer (histo)type (ICD-10 code): b) source of information (PR; HDR; DC); c) date of diagnosis, and whether a cancer has already been recorded. PRs, including gross and microscopic assessments, are considered the most reliable source of information, followed by HDRs, and then DCs.

The data considered for case registration purposes were judged “concordant” when they identified the same cancer site (i.e., up to the first three digits of the ICD-10 code). They were considered “compatible” when they referred to different sites within the same anatomo-functional district (e.g., lips, oral cavity and pharynx; see S1 Table).

For prevalent cancers, DP is used to compare the latest findings with previously obtained information. It distinguishes between primary and metachronous malignancies, that are recorded as new incident cases. Based on the available data (PRs, HDRs and DCs), DP differentiates between: a) incident cancers; b) prevalent cancers (already recorded), for which it updates the information in the incident case record); c) not cancers (to be excluded from the cancer registration process); d) cases requiring a dedicated RA (e.g., due to sources indicating discordant ICD-10 codes). Non cancer cases are further distinguished as neoplastic non-malignat lesions, and non-reoplastic lesions.

From among 20,485 cases of cancer registered in 2016, the present analysis considered 1,801 patients. For them to be included in the study, the following information had to be available: i) sex; ii) age group (0–59, 60–69, 70–79, 80+ years); iii) cancer status (incident *versus* prevalent); iv) available data sources; v) decision criteria applied during DP to identify cases (Table 1).

**Table 1:** Study sample size (1,801 out of 20,485 DP-cases registered in 2016) and proportions of cases (incident *versus* prevalent).

INCIDENT CANCERS					
Stratum	Sources	Decision criteria	Number of cases	Sample size	Proportion
01	PR, HDR, DC	PRs plus HDRs and/or DCs, all concordant	7,858	157	2%
02	PR, HDR, DC	PRs plus HDRs or DCs with metastases, ill-defined or unknown sites	1,730	87	5%
03	PR, HDR, DC	Two concordant PRs plus HDRs and/or DCs, compatible sites	848	298	35%
04	PR, HDR, DC	One PR plus HDRs and/or DCs, compatible sites	172	144	84%
05	HDR, DC	HDRs and DCs concordant, with discharge before terminal hospitalization	559	84	15%
06	HDR, DC	HDRs and DCs concordant, with only one terminal discharge	824	165	20%
07	PR	Two or more concordant PRs	353	106	30%
08	PR	Only one PR	1,106	221	20%
09	PR, HDR, DC	Metachronous cancer defined by random forest classifier	837	67	8%
01–09	Total incident cases		13,450	1,329	10%
**PREVALENT CANCERS**					
10	PR, HDR, DC	Cases defined as prevalent based on concordance rules	4,823	241	5%
11	PR, HDR, DC	Cases defined as prevalent based on compatibility rules	1,093	160	15%
12	PR, HDR, DC	Cases defined as prevalent based on concordance and compatibility rules	282	71	25%
10–12	Total prevalent cases		6,198	472	8%
**01–12**	**Total cases**		**20,485**	**1,801**	**9%**

Record(s) on which cancer diagnosis was based (PRs: pathology reports; HDRs: hospital discharge records; DCs: death certificates), and DP decision criteria. The total set of 20,485 cases identified by DP is divided into 12 strata: strata 01–09 concern incident cases in 2016 (strata 01–08 for new cases, and stratum 09 for metachronous cancers); strata 10–12 concern prevalent cases (new data recorded in 2016–2017 on cancers diagnosed already in 2015 were treated as cases of recurrent cancer).

We selected a proportional sample stratified by sex and age group. The sampling proportion for each stratum was chosen to ensure a 95% confidence interval, with a full consistency width of 0.5 when estimating the specific error rate for each of the 12 strata considered. The sampling proportion for strata 3 and 4 was readjusted to increase their reliability [7].

### Taking registrar-based cancer registration as the gold standard

The 1,801 cases considered were separately assigned to two registrars, who independently assessed each case, jointly reaching a conclusive decision based on their personal consultation of all available clinical charts (i.e., pathology, imaging and clinical-laboratory reports, etc.). For each case examined, the registrars established: a) the patient’s demographics, including their place of residence (municipality at the time of diagnosis); b) date of first cancer diagnosis (d/m/y); c) topography and morphology (coded according to ICD-O-3); d) information on which the cancer diagnosis had been established, ranked by reliability (from pathology and cytology, imaging and clinical pathology to clinical assessments, to DCs “only”).

### Comparing digital *versus* registrar-based cancer registration

The final RA dataset was then taken for reference in assessing the performance of digital registration, considering:

- the concordance between DP and RA findings on cancer status (incident / prevalent / not cancer), overall and by stratum;
- the concordance between DP and RA findings on cancer topography and morphology.

The level of concordance was ranked as an ordinal variable as follows:

- absent: RAs found no evidence of malignancy;
- low: evidence of malignancy, but discordant cancer topography (i.e., DP findings and RAs indicated ICD-10 codes belonging to different topographical groups; see S1 Table);
- intermediate: discordant anatomical site (first three digits of the ICD-10 code), but concordant topographical grouping (see S1 Table) [12];
- high: concordant topography and histology coding only affected by minor inconsistencies (histology groups based on ICD-O-3 codes are reported in S2 Table);
- full: concordant topography and morphology.

The absent, low and intermediate levels were considered as “unsatisfactory”, while the high and full concordance levels were taken to be “satisfactory”. The level of concordance was assessed overall and in relation to the following variables:

- stratum;
- number of sources (first three digits of the ICD-10 code for the site indicated in the RA):
  - 1 source: PR only;
  - 2 sources: HDR + PR or HDR + DC or PR + DC;
  - 3 sources: HDR + PR + DC;
- record(s) on which cancer diagnosis was based (microscopic, non-microscopic, DC “only”).

Differences in the date of first cancer diagnosis, and in a patient’s place of residence were also considered. The reliability of the DP approach was tested using Cohen’s kappa coefficient (K) [13], which adjusts the estimated level of concordance taking chance into account. The statistical analysis was performed using SAS, version 9.4 (SAS Institute, Cary, NC).

#### Ethics

Italian legislation identifies cancer registries as collectors of personal data for surveillance purposes with no need to obtain individuals’ explicit consent. Research ethics committee approval was not required for this study because it concerns a review of digital procedures routinely adopted by the Veneto Tumor Registry to identify cases of incident cancer, without any direct or indirect intervention on patients [14].

## Results

**Fig 1:**
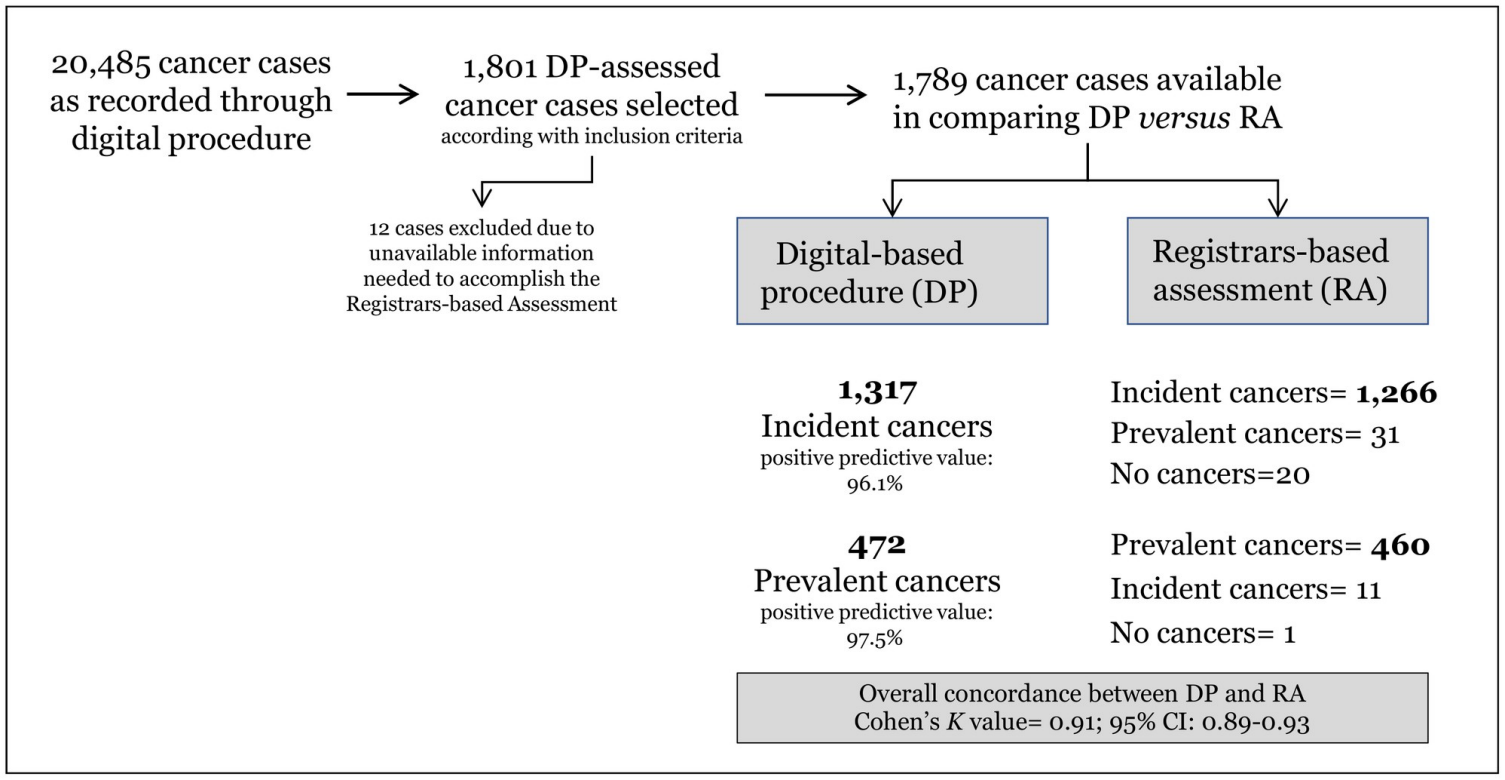
The study flowchart.

Twelve of the 1,801 cases examined were excluded because some of the information needed for the RA was missing (Fig 1). The final study sample thus consisted of 1,789 cases.

DP identified 1,317 cases of incident cancer, and 1,266/1,317 were confirmed by the corresponding RAs (positive predictive value [PPV] = 96.1%). The cancer status established by DP was not confirmed by the RAs in 51/1,317 cases (3.8%), 31 (2.4%) of which were reclassified as prevalent, and 20 (1.5%) as not cancer (Fig 1; Table 2).

**Table 2:** Level of concordance between digital processing (DP) and registrar-based assessments (RAs) of cases registered as incident and prevalent cancer.

	RA taken for reference			Total
	Incident	Prevalent	Not cancer	
**INCIDENT CASES (as defined by DP)**				
**Level of concordance**				
Absent	29 (2.2%)	0 (0.0%)	20 (1.5%)	**49** (3.7%) (95% CI: 2.8%-4.9%)
Low	18 (1.4%)	0 (0.0%)	0 (0.0%)	**18** (1.4%) (95% CI: 0.8%-2.2%)
Intermediate	133 (10.1%)	2 (0.2%)	0 (0.0%)	**135** (10.3%) (95% CI: 8.7%-12.0%)
High	472 (35.8%)	6 (0.5%)	0 (0.0%)	**478** (36.3%) (95% CI: 33.7%-39.0%)
Full	614 (46.6%)	23 (1.7%)	0 (0.0%)	**637** (48.4%) (95% CI: 45.6%-51.1%)
**Total**	**1266** (96.1%)	**31** (2.4%)	**20** (1.5%)	**1317** (100%)
**PREVALENT CASES (as defined by DP)**				
**Level of concordance**				
Absent	0 (0.0%)	1 (0.2%)	1 (0.2%)	**2** (0.4%) (0.1%-1.5%)
Low	0 (0.0%)	7 (1.5%)	0 (0.0%)	**7** (1.5% (0.6%-3.0%)
Intermediate	0 (0.0%)	36 (7.6%)	0 (0.0%)	**36** (7.6%) (5.4%-10.4%)
High	3 (0.6%)	91 (19.3%)	0 (0.0%)	**94** (19.9%) (16.4%-23.8%)
Full	8 (1.7%)	325 (68.9%)	0 (0.0%)	**333** (70.6%) (66.2%-74.6%)
**Total**	**11** (2.3%)	**460** (97.4%)	**1** (0.2%)	**472** (100%)

Cases assumed as non-concordant include not-cancer and non-malignant neoplasia. Percentages with 95% confidence intervals refer to total cases.

DP identified 472 cases of prevalent cancer, and 460 of them were confirmed by the RAs (PPV = 97.5%). Eleven (2.3%) of the remaining cases were reclassified as incident, and one (0.2%) as not cancer. Overall, the concordance between DP and RA findings exceeded 96% (“optimal” according to Cohen’s K [k-value = 0.91; 95% CI: 0.89–0.93]).

Among the incident cancers, the concordance level was absent, low, intermediate, full or high in 3.7%, 1.4%, 10.3%, 36.3% and 48.4% of cases, respectively. In 29 cases, what DP had identified as incident cancers were reclassified by the registrars as not cancer (no concordance). Among the prevalent cases identified by DP, there was no concordance in 2 cases (one reclassified by the registrars as non-neoplastic, the other as non-malignant tumor).

Among 21 patients reclassified by the registrars as not cancers (i.e. non-neoplastic), the sites involved were evenly distributed, while 11 of the 30 non-malignant tumors were *in situ* cutaneous melanomas. (S3 Table).

Stratifying the analysis by age group showed that nearly half of the cases in which there was no concordance concerned patients less than 60 years old (24/51; 47%). Table 3 shows the relationships between the levels of concordance and the criteria used to identify cases of cancer.

**Table 3:** Levels of concordance between the findings of digital processing and registrars’ assessments.

INCIDENT CANCERS (as defined by DP)						
	Level of concordance: Row Numbers and percentage (%)					
Stratum	Unsatisfactory			Satisfactory		Total
	Absent Numbert Number (%)	Low	Intermediate	High	Full concordance consistency	
01	7 (4.5)	2 (1.3)	4 (2.5)	57 (36.3)	87 (55.4)	157 (100)
02	0	1 (1.1)	1 (1.1)	33 (37.9)	52 (59.8)	87 (100)
03	2 (0.7)	6 (2.0)	111 (37.4)	75 (25.3)	103 (34.7)	297 (100)
04	1 (0.7)	0	0	92 (63.9)	51 (35.4)	144 (100)
05	0	1 (1.2)	3 (3.6)	39 (46.4)	41 (48.8)	84 (100)
06	5 (3.0)	2 (1.2)	8 (4.8)	30 (18.2)	120 (72.7)	165 (100)
07	5 (4.8)	2 (1.9)	0	55 (52.4)	43 (41.0)	105 (100)
08	28 (13.3)	4 (1.9)	7 (3.3)	79 (37.4)	93 (44.1)	211 (100)
09	1 (1.5)	0	1 (1.5)	18 (26.9)	47 (70.1)	67 (100)
**Total**	**49 (3.7)**	**18 (1.4)**	**135 (10.3)**	**478 (36.3)**	**637 (48.4)**	**1317 (100)**
**PREVALENT CANCERS (as defined by DP)**						
10	1 (0.4)	1 (0.4)	2 (0.8)	52 (21.6)	185 (76.8)	241 (100)
11	1 (0.6)	4 (2.5)	25 (15.6)	23 (14.4)	107 (66.9)	160 (100)
12	0	2 (2.8)	9 (12.7)	19 (26.8)	41 (57.7)	71 (100)
**Total**	**2 (0.4)**	**7 (1.5)**	**36 (7.6)**	**94 (19.9)**	**333 (70.6)**	**472 (100) (100)**

As expected, the risk of false-positive registrations was higher, the lower the number of variables considered (i.e., for a single PR, stratum 08, it was 13.3%).

The proportion of cases with a low level of concordance was much the same in all strata, ranging from 0% (strata 04 and 09) to 1.9% (strata 07 and 08). The proportions of cases with an intermediate concordance level were more diverse, with 37% of cases in stratum 03 (“Two concordant PRs plus HDRs and/or DCs, compatible sites”), while the percentages for the other strata ranged between 0% (Strata 04 and 07) and 4.8% (Stratum 06). A full concordance was found for 48.4% cases overall, and for more than 70% in Strata 06 and 09. The level of concordance was high or full for more than 90% of the cases of prevalent cancer.

### Concordance rate and sources of information

The concordance ranking correlated directly with the consistency of information obtained from the various sources. For the incident cancers, levels of concordance between the DP and RA findings were more likely to be unsatisfactory among cases classified on the strength of one (i.e., a PR), two or three sources of information (with proportions of 30.6%, 7.1% and 2.6%, respectively; see S4 Table). Similar results emerged for prevalent cancers, with unsatisfactory levels of concordance being a feature of cases registered on the strength of one (38.7%), two (4.7%), or three (2.5%) sources of information. The relationship between the number of sources and the level of concordance was significant (p <0.0001) according to Spearman’s correlation coefficient.

### Date of cancer diagnosis and place of residence

The date of first cancer diagnosis (d/m/y) registered by the DP and RAs was identical for 87.1% of patients; in the remainder, the difference was less than 30 days in 8.0% of cases, and more in 4.9%. The place of residence identified by the DP and RAs was consistent in 99.4% of the sample.

### Cancer topography and metachronous malignancies

After excluding the 51 cases reclassified by the registrars as false-positives (21 not cancers and 30 non-malignant tumors, i.e., concordance: absent), topography was assessed in 1,738 cases. DP and the RAs were consistent in identifying the cancer site (ICD-10) in 1,542/1,738 cases (88.7%, i.e., they were highly or fully concordant). The discrepancy involved different sites within the same anatomo-functional district in 171 cases (9.8%) (S5 Table), and different anatomo-functional district in 25 cases (1.4%) (S6 Table). Among the former 171 cases, the mismatch mainly involved colon *versus* rectal cancers. Among the latter 25 cases, in which different anatomo-functional districts were involved, DP registered 5 cases of cancer in the oral cavity (ICD10: C00-14) that the registrars reclassified as laryngeal cancers in 3 cases, and lip-skin tumors in 2 (and the level of concordance was consequently unsatisfactory). The registrars identified 19 metachronous cancers, none of which emerged from DP (see S7 Table).

## Discussion

This study quantified the consistency of cancer registrations obtained using DP with the assessments conducted by specialized registrars, which can be confidently considered as the gold standard. In the sample considered here, the level of concordance between the DP and RA findings exceeded 96%. This result not only supports the epidemiological reliability of digital cancer registration, but also shows that DP has the potential to cope with the growing need to provide oncologists (and patients’ communities) with timely information to facilitate the monitoring of newly-implemented cancer diagnosis and treatment protocols. This scenario demands a further shortening of the interval between initial cancer case registration and the availability of up-to-date information on cancer outcomes, which ultimately implies resorting increasingly to DP-based cancer registration.

Digital cancer registration can be affected by several issues [15, 16], including: i) the availability of reliable/detailed primary medical records; ii) the use of new diagnostic tests (such as molecular cancer profiling); iii) changes to diagnostic protocols (e.g., including the propensity to lower the number of invasive cancer assessments, i.e., histology); iv) new trends in cancer patient management (e.g., more outpatient treatments instead of inpatient care); v) the scattering of clinical data among different archives; and vi) an increasing amount of data to collect, resulting in a “big data” dimension [17]. The health care system where DPs are applied is also a crucial variable. In the setting of our study, the reliability of the clinical information is founded on at least two major strengths: a) a regional pathology data network (“AnaPath-flow”) that is currently the most dependable source of information on cancer incidence; b) the well-established informative value of HDRs, which can substantially help a cancer registry to monitor (and update) information on patient outcomes. The findings of the present study also underscore the importance of achieving these conditions.

This study provides unequivocal evidence of the amply satisfactory performance of DP methods for identifying and distinguishing between incident and prevalent cancers (with a misclassification rate of 5.1% for the former and 1.9% for the latter), with a negligible number of cases found to be false-positives.

Focusing in the present study on the different decision criteria applied using DP (by stratum and by number and type of sources of information) showed—as expected—that cancer cases were classified better when more complementary sources of information were available. False-positive cases were more common when only one source of information was available, and resulted mainly from coding inconsistencies. To improve coding reliability, periodical interdisciplinary meetings should promote both the empowering of pathologists in cancer coding and a pooling of registrars’ and pathologists’ clinical knowledge. Such interdisciplinary meetings have been formally included in the ISO certification [ISO9001:2015; certificate 24 09 2020 IT300700-1) of the registry where the present study was conducted.

In the sample considered here, DP overlooked 19 metachronous malignancies [18, 19]. It is worth emphasizing, however, that no pathology reports were available for any of these metachronous malignancies, which goes to show the pivotal role of pathology records in cancer registration. These cases most likely involved elderly cancer patients who had refused invasive diagnostics and/or patients treated before their cancer diagnosis had been established histologically.

Due to coding inconsistency, DP misclassified 11 incident cancers in our sample as prevalent cancers. The most prevalent topographic inconsistencies resulted from topographic coding errors (e.g., rectal disease misclassified as colon cancer; cancer involving the esophagogastric junction considered as either esophageal or gastric disease). *In situ* melanoma accounted for 37% of the false-positive cases registered using DP (*in situ* melanoma being classified as invasive due to inaccurate coding in pathology reports). All the above types of inconsistency mostly related to inappropriate coding in pathology reports, rather than to the digital process.

The present results are consistent with those obtained by a previous study on the same cancer registry, when the concordance between DP and RA findings was absent in 2.5% of cases and low in 3.4% (7). Applying different decision-making rules, the Ontario Cancer Registry [20, 21] found a prevalence of false-positives below 3.5%. Similar rates were obtained by registries established in South Wales [22], Eastern Scotland [23], and Scotland as a whole [24, 25], where the proportions of false-positive cases never exceeded 2%. These results are strongly influenced by the selection criteria adopted, however, and the prevalence of inconsistencies rose, the larger the number of variables considered: when only the first three digits of the ICD-10 code were considered, discrepancies between DP and RA findings dropped to 1.6% [26, 27].

### Strengths and limitations of this study

The main strengths of the present study lie in: i) having distinguished between incident and prevalent cancers in testing DP performance; ii) a large sample size, which ensured reliable error estimates; iii) a double-blinded study design, comparing DP versus real-world registrar-based assessments; and iv) the consensus-seeking procedure adopted in the registrar-based assessment.

The study’s main weakness lies in that the results relate to a specific health care system where the study was conducted (high level of the information network), theoretically limiting their generalizability to other settings.

## Conclusion

Automated case registration has a crucial role in ensuring the efficiency of cancer registries. The overall quality of such digital processing suffers from only trivial inconsistencies. Monitoring the efficiency of DP systems demands an interdisciplinary effort on the part of epidemiologists, oncologists, pathologists, registrars and statisticians. Their combined expertise enables specific problem areas to be identified and fixed. Quality control is a non-negotiable part of any digital cancer registration process.

## Supporting information

S1 Table Grouping of ICD-10 codes by anatomo-functional district^1^. ^1^ https://icd.who.int/browse10/2016/en%23/II.
(DOCX)

S2 Table Groups of malignant neoplasms considered histologically ‘different’ for the purpose of defining multiple tumors^2^. ^2^ https://www.encr.eu/sites/default/files/pdf/MPrules_july2004.pdf.
(DOCX)

S3 Table Distribution of ICD-10 codes assigned by the digital procedure (DP) to false-positive cases (non-cancers or not malignant): Absent concordance. (DOCX)

S4 Table Concordance between digital procedure (DP) and registrar-based assessment (ReA) of incident and prevalent cases, by number of sources. (DOCX)

S5 Table Distribution of ICD-10 codes for patients with different sites within the same anatomo-functional districts, as assigned by the digital procedure (DP) and registrar-based assessment (RA): Intermediate concordance. Only the most common combinations (cumulative 75%) are listed.
(DOCX)

S6 Table Distribution of ICD-10 codes for cancer cases involving different topographical group, as assigned by digital processing (DP) and registrar-based assessments (RA): Low concordance. (DOCX)

S7 Table Distribution of ICD-10 codes for (unregistered) metachronous cancers. (DOCX)
