# Supplementary material for: Quality control on digital cancer registration
Source: PLoS One. 2022 Dec 22;17(12):e0279415. doi: 10.1371/journal.pone.0279415 (PMC9778557; doi:10.1371/journal.pone.0279415)
Supplement: S1 Table — 1 https://icd.who.int/browse10/2016/en%23/II. (DOCX) [file pone.0279415.s001.docx]

**S1 Table.** Grouping of ICD-10 codes by anatomy-functional district ^1^

| **ICD-10 codes** | **Description** |
| --- | --- |
| C00-C14 | Lips, oral cavity, pharynx |
| C15-C26, C48 | Digestive system and peritoneum |
| C30-34, C37-39, C45 | Respiratory system |
| C40-C41 | Bone |
| C47-C49 | Connective and soft tissue |
| C43-C44 | Skin |
| C50 | Breast |
| C51-C58 | Female genital organs |
| C60-63 | Male genital organs |
| C64-C68, D09.0, D41.4 | Urinary system |
| C69 | Eye |
| C70-C72 | Brain and nervous system |
| C73-C75 | Thyroid and other endocrine glands |
| C81-C86; C88.4 | Lymphomas |
| C88 (excluding C88.4)-C90 | Myeloma and immunoproliferative diseases |
| C91-C95 | Leukemia |
| C46 | Kaposi's sarcoma |
| C80 | Malignant neoplasm without specification of site |
| D45, D46, D47 | Tumors of uncertain behavior of other and unspecified sites |

^1^<https://icd.who.int/browse10/2016/en#/II>
