# Supplementary material for: Quality control on digital cancer registration
Source: PLoS One. 2022 Dec 22;17(12):e0279415. doi: 10.1371/journal.pone.0279415 (PMC9778557; doi:10.1371/journal.pone.0279415)
Supplement: S2 Table — 2 https://www.encr.eu/sites/default/files/pdf/MPrules_july2004.pdf. (DOCX) [file pone.0279415.s002.docx]

**S2 Table.** Groups of malignant neoplasms considered to be histologically ‘different’ for the purpose of defining multiple tumours.^2^

| **Morphology group** | **ICD-O-3 codes** |
| --- | --- |
| Carcinomas | |
| 1. Squamous and transitional cell carcinoma | 8051-8084, 8120-8131 |
| 1. Basal cell carcinomas | 8090-8110 |
| 1. Adenocarcinomas | 8140-8149, 8160-8162, 8190-8221, 8260- 8337, 8350-8551, 8570-8576, 8940-8941 |
| 1. Other specific carcinomas | 8030-8046, 8150-8157, 8170-8180, 8230- 8255, 8340-8347, 8560-8562, 8580-8671 |
| 1. Unspecified carcinomas(NOS) | 8010-8015, 8020-8022, 8050 |
| 1. Sarcomas and soft tissue tumours | 8680-8713, 8800-8921, 8990-8991, 9040- 9044, 9120-9125, 9130-9136, 9141-9252, 9370-9373, 9540-9582 |
| 1. Mesothelioma | 9050-9055 |
| Tumours of haematopoietic and lymphoid tissues | |
| 1. Myeloid | 9840, 9861-9931, 9945-9946, 9950, 9961- 9964, 9980-9987 |
| 1. B-cell neoplasms | 9670-9699, 9728, 9731-9734, 9761-9767, 9769, 9823-9826, 9833, 9836, 9940 |
| 1. T-cell and NK-cell neoplasms | 9700-9719, 9729, 9768, 9827-9831, 9834, 9837, 9948 |
| 1. Hodgkin lymphoma | 9650-9667 |
| 1. Mast-cell Tumours | 9740-9742 |
| 1. Histiocytes and Accessory Lymphoid cells | 9750-9758 |
| 1. Unspecified types | 9590-9591, 9596, 9727, 9760, 9800- 9801, 9805, 9820, 9832, 9835, 9860, 9960, 9970, 9975, 9989 |
| 1. Kaposi sarcoma | 9140 |
| 1. Other specified types of cancer | 8720-8790, 8930-8936, 8950-8983, 9000- 9030, 9060-9110, 9260-9365, 9380- 9539 |
| 1. Unspecified types of cancer | 8000-8005 |

^2^ <https://www.encr.eu/sites/default/files/pdf/MPrules_july2004.pdf>
