# Supplementary material for: Quality control on digital cancer registration
Source: PLoS One. 2022 Dec 22;17(12):e0279415. doi: 10.1371/journal.pone.0279415 (PMC9778557; doi:10.1371/journal.pone.0279415)
Supplement: S3 Table — (DOCX) [file pone.0279415.s003.docx]

**S3 Table.** Distribution of ICD-10 codes assigned by the digital procedure (DP) to false-positive cases (non-cancers or not malignant): absent concordance.

| **Not cancer** | | |  |  | **Not malignant** | | |
| --- | --- | --- | --- | --- | --- | --- | --- |
| ICD-10 | N | % |  |  | ICD-10 | N | % |
| C67.9 Bladder | 3 | 14.3 |  |  | C43 Melanoma os skin | 11 | 36.6 |
| C17 Small intestine | 3 | 14.3 |  |  | C53.9 Cervix uteri | 4 | 13.3 |
| C61 Prostate | 2 | 9.5 |  |  | C50.9 Breast | 3 | 10.0 |
| C18.9 Colon | 1 | 4.8 |  |  | C34.9 Lung | 2 | 6.7 |
| C22.7 Liver | 1 | 4.8 |  |  | C49.9 Soft tissue | 2 | 6.7 |
| C24.9 Other Biliary tract | 1 | 4.8 |  |  | C00.9 Lip | 1 | 3.3 |
| C34.9 Lung | 1 | 4.8 |  |  | C22.9 Liver | 1 | 3.3 |
| C43.6 Melanoma ok skin | 1 | 4.8 |  |  | C25.9 Pancreas | 1 | 3.3 |
| C50.9 Breast | 1 | 4.8 |  |  | C54.1 Corpus uteri | 1 | 3.3 |
| C53.9 Cervix uteri | 1 | 4.8 |  |  | C56 Ovary | 1 | 3.3 |
| C68.9 Urinary organs | 1 | 4.8 |  |  | C64 Kidney | 1 | 3.3 |
| C73 Thyroid | 1 | 4.8 |  |  | C83.3 Diffuse large B-cell lymphoma | 1 | 3.3 |
| C75.1 Other endocrine glands | 1 | 4.8 |  |  | C85.9 Non-Hodgkin’s lymphoma | 1 | 3.3 |
| C81.9 Hodgkin’s lymphoma | 1 | 4.8 |  |  |  |  |  |
| C85.9 Non hodgkin’s lymphoma | 1 | 4.8 |  |  |  |  |  |
| D46.9 Myelodysplastic syndrome | 1 | 4.8 |  |  |  |  |  |
|  |  |  |  |  |  |  |  |
| **Total** | **21** | **100** |  |  | **Total** | **30** | **100** |
