# Supplementary material for: Quality control on digital cancer registration
Source: PLoS One. 2022 Dec 22;17(12):e0279415. doi: 10.1371/journal.pone.0279415 (PMC9778557; doi:10.1371/journal.pone.0279415)
Supplement: S4 Table — (DOCX) [file pone.0279415.s004.docx]

**S4 Table.** Concordance between digital procedure (DP) and registrar-based assessment (ReA) of incident and prevalent cases, by number of sources.

| **INCIDENT CASES (as defined by the DP)** | | | | | | | |
| --- | --- | --- | --- | --- | --- | --- | --- |
| **Number of**  **concordant sources** |  | **Level of concordance** | | | | | **Total** |
|  |  | **Absent** | **Low** | **Intermediate** | **High** | **Full**  **concordance** |  |
| 1 | N | 35 | 13 | 100 | 157 | 178 | 483 |
|  | % | 7.2 | 2.7 | 20.7 | 32.5 | 36.9 | 100 |
| 2 | N | 14 | 5 | 32 | 292 | 376 | 719 |
|  | % | 1.9 | 0.7 | 4.5 | 40.6 | 52.3 | 100 |
| 3 | N | 0 | 0 | 3 | 29 | 83 | 115 |
|  | % | 0.0 | 0.0 | 2.6 | 25.2 | 72.2 | 100 |
| **PREVALENT CASES (as defined by the DP)** | | | | | | | |
| **Number of**  **concordant sources** |  | **Level of concordance** | | | | | **Total** |
|  |  | **Absent** | **Low** | **Intermediate** | **High** | **Full** |  |
| 1 | N | 1 | 5 | 23 | 7 | 39 | 75 |
|  | % | 1.3 | 6.7 | 30.7 | 9.3 | 52.0 | 100 |
| 2 | N | 1 | 2 | 10 | 60 | 203 | 276 |
|  | % | 0.4 | 0.7 | 3.6 | 21.7 | 73.6 | 100 |
| 3 | N | 0 | 0 | 3 | 27 | 91 | 121 |
|  | % | 0.0 | 0.0 | 2.5 | 22.3 | 75.2 | 100 |
