# Supplementary material for: Quality control on digital cancer registration
Source: PLoS One. 2022 Dec 22;17(12):e0279415. doi: 10.1371/journal.pone.0279415 (PMC9778557; doi:10.1371/journal.pone.0279415)
Supplement: S5 Table — Only the most common combinations (cumulative 75%) are listed. (DOCX) [file pone.0279415.s005.docx]

**S5 Table.** Distribution of ICD-10 codes for patients with different sites within the same anatomo-functional districts, as assigned by the digital procedure (DP) and registrar-based assessment (RA): intermediate concordance. Only the most common combinations (cumulative 75%) are listed.

| **ICD-10**  **assigned by DP** | **ICD-10**  **assigned by ReA** | **N** | **%** |
| --- | --- | --- | --- |
| C18 Colon | C19, C20 Rectum | 55 | 32.7 |
| C55 Uterus, not specified | C54 Corpus uteri | 13 | 7.7 |
| C15 Oesophagus | C16 Stomach | 11 | 6.5 |
| C16 Stomach | C15 Oesophagus | 8 | 4.8 |
| C39 Other respiratory organs | C34 Lung | 6 | 3.6 |
| C53 Cervix uteri | C54 Corpus uteri | 6 | 3.6 |
| C14 Other oral cavity | C11 Nasopharynx | 5 | 3.0 |
| C38 Heart, mediastinum, pleura | C34 Lung | 4 | 2.4 |
| C55 Uterus, not specified | C53 Cervix uteri | 4 | 2.4 |
| C06 Other parts of mouth | C00 Lip | 3 | 1.8 |
| C38 Heart, mediastinum, pleura | C45 Mesothelioma | 3 | 1.8 |
| C54 Corpus uteri | C53 Cervix uteri | 3 | 1.8 |
| C64 Kidney | C65 Renal pelvis | 3 | 1.8 |
| C67 Bladder | C68 Other urinary organs | 3 | 1.8 |
