# Supplementary material for: Quality control on digital cancer registration
Source: PLoS One. 2022 Dec 22;17(12):e0279415. doi: 10.1371/journal.pone.0279415 (PMC9778557; doi:10.1371/journal.pone.0279415)
Supplement: S6 Table — (DOCX) [file pone.0279415.s006.docx]

**S6 Table.** Distribution of ICD-10 codes for cancer cases involving a different topographical group, as assigned by the digital procedure (DP) and registrar-based assessment (RA): low concordance.

| **ICD-10 assigned by DP** | **ICD-10 assigned by ReA** | **N** |
| --- | --- | --- |
| C61 Prostate | C67, D09.0 Bladder | 5 |
| C32 Larynx | C12 Pyriform sinus | 3 |
| C22 Liver | C80 Malignant neoplasm without specification of site | 3 |
| C00 Lip | C44 Skin other than melanoma | 2 |
| C85 Non-Hodgkin’s lymphoma | C91 Lymphoid leukaemia | 2 |
| C02 Base of tongue | C32 Larynx | 1 |
| C06 Other parts of mouth | C32 Larynx | 1 |
| C14 Other oral cavity | C32 Larynx | 1 |
| C18 Colon | C80 Malignant neoplasm without specification of site | 1 |
| C22 Liver | C34 Lung | 1 |
| C33 Trachea | C80 Malignant neoplasm without specification of site | 1 |
| C34 Lung | C80 Malignant neoplasm without specification of site | 1 |
| C49 Soft tissue | C44 Skin other than melanoma | 1 |
| C50 Breast | C44 Skin other than melanoma | 1 |
| C83 Diffuse non-Hodgkin’s lymphoma | C88.0 Waldenström macroglobulinemia | 1 |
| **Total** | | **25** |
