# Supplementary material for: Quality control on digital cancer registration
Source: PLoS One. 2022 Dec 22;17(12):e0279415. doi: 10.1371/journal.pone.0279415 (PMC9778557; doi:10.1371/journal.pone.0279415)
Supplement: S7 Table — (DOCX) [file pone.0279415.s007.docx]

**S7 Table.** Distribution of ICD-10 codes for (unregistered) metachronous cancers.

| **Found among INCIDENT cases** | | | **Found among PREVALENT cases** | | |
| --- | --- | --- | --- | --- | --- |
| **ICD-10** | **N** | **%** | **ICD-10** | **N** | **%** |
| C61 Prostate | 3 | 37.5 | C61 Prostate | 5 | 45.5 |
| C17 Small intestine | 1 | 12.5 | C64 Kidney | 2 | 18.2 |
| C20 Rectum | 1 | 12.5 | C16 Stomach | 1 | 9.1 |
| C54 Corpus uteri | 1 | 12.5 | C44 Skin other than melanoma | 1 | 9.1 |
| C56 Ovary | 1 | 12.5 | C80 Malignant neoplasm without specif. of site | 1 | 9.1 |
| C65 Renal pelvis | 1 | 12.5 | D41.4 Neoplasm of uncertain behavior of bladder | 1 | 9.1 |
| **Total** | **8** | **100** | **Total** | **11** | **100** |
